# Older people receiving family‐based support in the community: a survey of quality of life among users of ‘Shared Lives’ in England

**DOI:** 10.1111/hsc.12422

**Published:** 2017-01-29

**Authors:** Lisa Callaghan, Nadia Brookes, Sinead Palmer

**Affiliations:** ^1^ Personal Social Services Research Unit University of Kent Canterbury UK

**Keywords:** community‐based support, older people, personalisation, quality of life, Shared Lives

## Abstract

Shared Lives (adult placement) is a model of community‐based support where an adult who needs support and/or accommodation moves into or regularly visits the home of an approved Shared Lives carer, after they have been matched for compatibility. It is an established but small service which has been used mainly by people with learning disabilities but which has the potential to offer an alternative to traditional services for some older people. However, there is little research on the outcomes for older users of Shared Lives. This paper presents findings from a survey of 150 older people using Shared Lives support across 10 Shared Lives schemes in England, which took place between June 2013 and January 2014. The aim was to identify outcomes for older users of Shared Lives and compare these to outcomes for older users of other social care services. In the absence of an ideal study design involving randomised allocation, statistical matching was used to generate a comparison group from the Adult Social Care Survey from 2011/12, with 121 cases matched to 121 Shared Lives cases. The main outcome measures were Social Care‐Related Quality of Life (measured by the ASCOT) and overall quality of life. Findings indicated that Shared Lives can deliver good outcomes for older people, particularly for overall quality of life. In comparison to the matched group of older people using other forms of support, there was some evidence that Shared Lives may deliver better outcomes in some aspects of quality of life. Limitations to the research mean, however, that more work is needed to fully understand the role Shared Lives could play in supporting older people.

**What is known about this topic**

- Shared Lives support is mainly used by people with learning disabilities.
- There is some evidence of high levels of satisfaction among users and Shared Lives is rated highly by the Care Quality Commission in England.
- Shared Lives has been identified as a potential option for older people.

**What this paper adds**

- Shared Lives appears to deliver good outcomes for older people.
- In comparison to a matched group of older people using other forms of social care, Shared Lives users reported better quality of life in some areas.
- Shared Lives should be included as part of support options offered to older people and their families.

## Introduction

At a time of substantial cuts to social care spending and an ageing population, policy makers and practitioners must ascertain how best to provide for the care and support needs of older people. Central to current social care policy in the UK and Western Europe is the personalisation agenda with an emphasis on giving people active choice and control over their care and support (DH, 2010, 2012). Additionally, in recent years, councils in England have been encouraged to reduce inappropriate admissions to residential care through improving options for community‐based care and support (DH, 2010) and changing their focus to prevention and early intervention (Care Act, 2014). Older people have been identified as among those hard to include in the move to personalisation (Newbronner *et al*. 2011), and concern has been expressed regarding the effectiveness of home care services for delivering personalised care and support for this group (Equality and Human Rights Commission, 2011). The challenge of combating loneliness and social isolation, to which people can be particularly vulnerable in older age (Windle *et al*. 2011) is not easily met by these services, particularly in times of increasing budget cuts. It has been noted that as people age quality of life is particularly shaped by the relationships they have and communities they live in (Blood 2013), and that attachment to place and a sense of belonging to the physical and social environment may increase (Lawton 1985, Gilleard *et al*. 2007). In recognition of these factors, practitioners, commissioners, service users and carers need evidence on alternative, community‐based service options for older people.

Scoping research funded by the National Institute for Health Research (NIHR) School for Social Care Research (SSCR) highlighted Shared Lives (previously known as adult or family placement) as a personalised service mainly used by people with learning disabilities, which may have the potential to deliver good outcomes for older people (Callaghan *et al*. 2012). This paper draws on a follow‐on study which aimed to generate evidence about the use of Shared Lives (henceforth noted as SL) for older people. It presents findings from a national survey of older people using SL, describing quality of life outcomes. Outcomes are compared with a matched sample of older people using other forms of support. Following this, implications of the findings are discussed.

### Shared lives

Adult or family placement‐type services have been used for many years with different client groups and in most parts of the world, particularly Northern Europe and the USA, although terminology and definitions vary (Schofield 2009). The oldest formally constituted service is in Geel, Belgium where people with mental health conditions and learning disabilities have been supported in local families for centuries, while in the UK the Liverpool Personal Service Society has been providing adult placements since the 1970s (Fiedler 2005). There was a growth in adult placements in the UK during the 1970s following the setting up of local authority social service departments and a policy shift away from institutional care towards care in the community (Schofield 2009), and by the 1980s many schemes mainly offered long‐term care and support for people with learning disabilities (Dagnan 1997).

Today, SL is the term used to describe family‐based support where service users are included in the family and community life of a ‘Shared Lives carer’, who uses their family home as a resource (see Box 1 for a detailed definition). There are different types of SL arrangement: residential or long term; respite or short breaks; day support; rehabilitative or intermediate support; and outreach support (which incorporates elements of the SL model, but is delivered in the service user's home). SL carers are recruited, trained and approved by local schemes, and are paid a fixed amount rather than an hourly rate. They are carefully matched to the person requiring support to ensure compatibility, a crucial element of the SL model. The key difference between SL and other community‐based models, such as Supported Living, lies in the importance placed on integration with a family and mutuality of relationships.

Box 1 **Detailed definition of Shared Lives**

**Table:** 

*Shared Lives is a service provided by individuals and families in local communities and is distinguished by the following features:*
Arrangements are part of organised Shared Lives Schemes that approve and train Shared Lives Carers, receive referrals, match the needs of service users with Shared Lives Carers, and monitor the arrangements.
People using Shared Lives services have the opportunity to be part of the Shared Lives Carer's family and social networks.
Shared Lives Carers use their family home as a resource.
Arrangements provide committed and consistent relationships.
The relationship between the Shared Lives Carer and the person placed with them is of mutual benefit.
Shared Lives Carers can support up to three people at any one time (up to two people in Wales).
Shared Lives carers do not employ staff to provide care to the people placed with them.

Until recently, the extent of the use of SL support in the UK, and by whom, was unknown. Data from a survey of SL schemes in England conducted by Shared Lives Plus (the UK network for family‐based and small‐scale ways of supporting adults) were used to estimate that over 9660 people were supported by SL at the end of 2013, 1600 of whom were older people. This includes older people who are receiving support from SL for other reasons such as a learning disability or physical impairment. Overall, it was projected that around 7% of the total number of people receiving SL support had dementia or were frail older people (Shared Lives Plus, 2014). As such, SL represents a small proportion of adult care and support services, although the sector is growing (Shared Lives Plus, 2016).

SL has strong advocates, and has been compared favourably to other care and support options on some key indicators of personalisation, such as inclusion, flexibility, choice and control (NAAPS, 2010). It is consistently rated highly by the Care Quality Commission (the regulator for health and social care services in England) and in 2013/2014 was rated as achieving 100% compliance with quality standards in relation to respect and involvement (Care Quality Commission, 2014).

There has been relatively little research on SL, although there is some evidence of high levels of satisfaction among users (Fiedler 2005, NAAPS and IESE, 2009). Research studies have suggested that users value being treated as an individual, being part of a family and taking part in household tasks (Ware 1987, Dagnan & Drewett 1988, Robinson & Simons 1996). As part of the Shared Lives Plus survey of SL schemes, a survey of 80 SL carers indicated that SL arrangements had helped users (including older people) develop independent living skills and increase their social participation (Shared Lives Plus, 2014). There has been little attempt to compare outcomes of SL support to other forms of care (Dagnan 1997, Schofield 2009). Although the majority of SL support in the UK is for people with learning disabilities, it has been identified as having potential for older people, including those with dementia (Valios 2010, Fox 2011, Bell & Litherland 2013). SL has been proposed as a possible alternative to traditional respite care (McConkey *et al*. 2002, Valios 2010) and may also provide an alternative to moving to a care home for some. There is some indication that SL schemes and local authorities would support expansion of their services to include more older people (Brookes & Callaghan 2013). However, there is a lack of robust evidence about outcomes for older people using SL, and how these may compare to older people using alternative care and support. To begin to address this, the SSCR commissioned the Personal Social Services Research Unit at the University of Kent to examine the potential of SL for older people.

### Methods

#### The study

The findings reported here draw on a survey of older people using SL support in England. The survey was part of a wider study conducted between January 2012 and April 2014, which collected information on the outcomes and experiences of older people using SL and their family carers, issues for implementation and expansion of SL schemes to support older people, perceived demand for SL from older people and costs.

The study had ethics approval from the Social Care Research Ethics Committee, support from the Association of Directors of Adult Social Services and research governance approval from participating councils. It benefited from the guidance of national and local project advisory groups involving practitioners, SL users and carers, family carers and academics.

### Data collection

The aim of the survey was to identify the outcomes of older users of SL and compare those outcomes to older users of other social care services. In the absence of an ideal study design involving randomised allocation, statistical matching (described below) was used to generate a comparison group from the Adult Social Care Survey (ASCS) from 2011/2012.

A self‐completion questionnaire was developed using questions from the ASCS and other existing surveys to facilitate comparison, alongside a number of demographic questions (see Box 2). An ‘easy‐read’ version of the questionnaire was developed based on the easy‐read ASCS, and was made available to anyone who preferred this format. The main outcome measure used was the Adult Social Care Outcomes Toolkit (ASCOT) (Netten *et al*. 2012), designed to measure aspects of quality of life specifically relating to social care and applicable across different care settings, with all user groups (http://www.pssru.ac.uk/ascot). The eight domains of social care‐related quality of life (SCRQoL) that the ASCOT measure draws on are personal cleanliness and comfort; food and drink; accommodation cleanliness and comfort; safety; control over daily life; social participation and involvement; occupation; and dignity. Items are coded to represent the ‘ideal’ state, having no needs, some needs or high needs in each domain (see Box 3). To create an overall SCRQoL ‘score’, responses are weighted to reflect the relative importance of each domain and level of need, based on previous work on the preferences of social care users and the general population, estimated using a combination of Best‐Worst Scaling and Time Trade‐Off approaches (Potoglou *et al*. 2011, Netten *et al*., 2003, pp. 85–87). These are then summed to give a total ranging from −0.17 to 1.00, with 0 equating to ‘being dead’ and 1.00 to an ‘ideal’ state; scores less than 0 refer to a state that is rated as being worse than death (Netten *et al*., 2003).

Box 2 **Items and measures included in questionnaire^a^**

**Table:** 

Background information	Age, gender, ethnicity
	Type of SL support
Health and dependency	Self‐perceived health (Robine *et al*. 2002), 5‐point scale
	Ability to perform activities of daily living (ADLs) and instrumental activities of daily living (IADLs), eight individual items, scores summed to give dependency score from 0–16
	Two items from the EQ5D (Euro‐QoL^b^) which measures health‐related quality of life: extent of pain/discomfort, extent of anxiety/depression. 3‐point scale
Assistance with questionnaire	Level of help (single question)
	Source of help (single question)
Outcome measures	Overall quality of life (Bowling 1995); 7‐point scale, collapsed into 5 points to be consistent with easy‐read version
	Social care‐related quality of life (measured using the ASCOT; Netten *et al*. 2012)

a The easy‐read version of the questionnaire had fewer items; for the purposes of this paper, only measures included in both versions are reported.

b ^©^1990 EuroQol Group. EQ‐5D™ is a trademark of the EuroQol Group.

Box 3 **Meaning of response options**

**Table:** 

Ideal	The preferred situation, in which needs are met to the desired level
No needs	Where needs are met, but not to the desired level
Low‐level needs	Where there are needs, but these do not have an immediate or long‐term health implication
High‐level needs	Where there are needs and these have an immediate or long‐term health implication

SL schemes were recruited through a scoping survey in July 2012 and a follow‐up email from Shared Lives Plus. All schemes providing services to older people were asked to volunteer, and 12 agreed. Managers of the schemes were sent documentation relating to the survey: invitation letters for participants including information about the project and what participation would involve, questionnaires and envelopes for their return. As numbers of older people using SL were expected to be small, schemes were asked to send these to all eligible users of their service, and to return completed surveys to the researchers. Following advice from the advisory groups, a broad inclusion criteria were used; older people (age 65 and over), including people with learning disabilities, and using any form of SL support (including outreach delivered in the service user's own home). Only individuals with capacity to consent and to understand the questions were included, a judgement made by the SL workers distributing the questionnaire based on their knowledge of the service users in their scheme. Schemes were sent questionnaires corresponding to the number of eligible service users they had (ranging from 3 to over 200), a total of 430. Data collection took place between June 2013 and January 2014.

### Data analysis

Descriptive statistics were used to describe the SL sample. For comparisons between participants using different types of SL, chi‐squared (χ^2^) tests were used for variables with unordered categories. Kruskal–Wallis or Mann–Whitney tests were computed for variables with small numbers of ordered categories. When the variable was continuous or had more than five meaningfully ordered categories, t‐tests or ANOVA were used. Posthoc comparisons (the Bonferroni comparison) were used where appropriate to compare differences between groups. Multivariate analyses were used to explore factors associated with variations in outcome in the SL sample. Screening of the data indicated that it did not meet a number of assumptions needed to perform ordinary least squares (OLS) multiple regression, and so regression with robust standard errors (Chen *et al*., 2003) was performed. Selection of variables for inclusion was based on their relationship with the outcome variable and with other variables of interest such as type of SL. The small sample size, however, meant that this was limited to a relatively small number of explanatory variables (Tabachnick & Fidell 2007). Only complete cases were included in the regression models. Just 3% of the sample was missing the overall quality of life score but 15 people were missing a SCRQoL score due to missing data on one or more items. A logistic regression model was computed using the outcome variable as a dummy variable to indicate ‘missing’ or ‘not missing’ the SCRQOL score. The model was non‐significant, suggesting that those missing a SCRQoL score did not differ significantly from those who did not in terms of background characteristics, and so imputation of missing data was not deemed necessary.

Tests for difference between the SL sample and the ASCS sample were computed. Given that the two samples were different, propensity score matching (PSM) (Rosenbaum & Rubin 1983, Austin 2011) was used to generate a matched sample, adjusting for baseline imbalances in the two groups and so facilitate an unbiased comparison of outcomes. PSM is a non‐experimental method of sampling that produces a ‘control group’ whose distribution of variables is similar to that of the study group. A significance level of 0.05 was adopted for all tests. Data were analysed using Stata SE13.

## Results

### Participants

The sample comprised 150 older users of SL, a response rate of 29% of the total number of questionnaires requested by schemes (it was not possible to identify how many were actually sent out) and approximately 9% of the total number of older people estimated by Shared Lives Plus to be using SL at the time of the survey.

Half of the sample were female. The age of respondents ranged from 65 to 102, with an average age of 77 (SD = 8.68). The vast majority (98%) of the sample was white British, with one Asian/Asian British and two black/black British respondents.

Questionnaires were returned from 10 SL schemes, with a range of 1–64 per scheme. Four ‘types’ of SL were represented: long‐term/residential, day support, respite/short breaks and outreach support (see Table 1). Long‐term SL users were spread across eight schemes, while the majority (89%) of day support users came from only one scheme. Respite was used by the smallest number of people. The outreach users made up a significant proportion of the sample and came from one scheme. Although this service is not traditionally considered as SL, the matching process remains the same, meaning that it is possible to attain the same level of personalisation as in ‘traditional’ SL services. Forty people completed the easy‐read version of the questionnaire. Thirty‐six of these were using long‐term SL, reflecting the tendency for long‐term placements to be used by people with a learning disability.

**Table 1:** Responses by type of SL arrangement

Type of arrangement	No. of schemes	No. of responses (% of sample)
Long term/residential	8	52 (34.7)
Day support	2	35 (23.3)
Respite/short breaks	3	8 (5.3)
Outreach/kinship support	1	55 (36.7)
Total		150

Table 2 shows the differences between individuals using different types of SL arrangement. There was a broad range of ages across all groups. Tests indicated that those using long‐term SL were younger than those using day support or outreach. They were also less dependent, reported experiencing less pain or discomfort, less anxiety and depression and rated their health as better. There was no significant difference in proportions of males and females.

**Table 2:** Characteristics of older people using different types of SL support

	Long term	Day support	Respite/short breaks	Outreach	Missing	Test	*P*
	*n* (%)	*n* (%)	*n* (%)	*n* (%)			
Age							
Range	65–93	65–96	68–91	66–102			
Mean (SD)	71.6 (5.81)	78.3 (9.49)	77.9 (9.13)	81.3 (7.79)	10	*F* (3,136) = 13.65	<0.001^a^
Gender							
Female	26 (51.0)	19 (57.6)	2 (25.0)	24 (47.1)	7		0.419^b^
Male	25 (49.0)	14 (42.4)	6 (75.0)	27 (52.9)			
Dependency (ADL score)^b^							
Range	0–7	0–16	2–7	0–16			
Mean (SD)	1.84 (1.85)	5.44 (4.12)	4.43 (2.07)	7.23 (4.26)	14	*F* (3,132) = 19.27	<0.001^a^
Pain/discomfort^c^							
Mean (SD)	1.44 (0.54)	1.76 (0.65)	1.85 (0.90)	1.94 (0.53)	3		
None	30 (57.7)	12 (35.3)	3 (42.9)	9 (16.7)		χ^2^(3) = 19.03^d^	<0.001
Moderate	21 (40.4)	18 (52.9)	2 (28.6)	39 (72.2)			
Extreme	1 (1.9)	4 (11.8)	2 (28.6)	6 (11.1)			
Anxiety/depression^c^							
Mean (SD)	1.33 (0.51)	1.68 (0.53)	1.75 (0.46)	1.94 (0.61)	4		
None	36 (69.2)	12 (35.3)	2 (25.0)	16 (30.8)		χ^2^(3) = 18.91^d^	0.001
Moderate	15 (28.9)	21 (61.8)	6 (75.0)	31 (59.6)			
Extreme	1 (1.9)	1 (2.9)	0 (0.0)	5 (9.6)			
Self‐perceived health							
Mean (SD)	1.90 (0.85)	2.65 (0.77)	3.50 (1.07)	3.02 (0.91)	3	*F* (3,143) = 18.16	<0.001
Very good	19 (36.5)	3 (8.8)	0 (0.0)	1 (1.9)			
Good	21 (40.4)	9 (26.5)	2 (25.0)	13 (24.9)			
Alright	10 (19.2)	19 (55.9)	1 (12.5)	28 (52.8)			
Bad	2 (3.9)	3 (8.8)	4 (50.0)	6 (11.3)			
Very bad	0 (0.0)	0 (0.0)	1 (12.5)	5 (9.4)			
Length of time using SL							
<1 year	2 (4.1)	4 (13.8)	1 (12.5	20 (41.7)		χ^2^(3) = 52.92^d^	<0.001
>1 year but <3 years	5 (10.2)	13 (44.8)	2 (25.0)	20 (41.7)			
>3 years but <5 years	9 (18.4)	3 (10.3)	2 (25.0)	6 (12.5)			
>5 years but <10 years	16 (32.7)	7 (24.1)	0 (0.0)	2 (4.2)			
>10 years but <15 years	7 (14.3)	0 (0.0)	0 (0.0)	0 (0.0)			
>15 years but <20 years	2 (4.1)	1 (3.5)	1 (12.5)	0 (0.0)			
20 years or more	8 (16.3)	1 (3.5)	2 (25.0)	0 (0.0)			
Help with survey completion							
No help	9 (17.3)	6 (18.8)	1 (14.3)	2 (3.8)	6		0.001^b^
Assistance	43 (82.7)	20 (62.5)	5 (71.4)	41 (77.4)			
Proxy	0 (0.0)	6 (18.8)	1 (14.3)	10 (18.9)			

a Bartlett's test of equal variances was statistically significant.

b Fisher's exact test was used, therefore no accompanying test statistic.

c 0–16, higher scores indicate greater dependency. 0–3, higher scores indicate greater depression/anxiety, greater pain/discomfort.

d Kruskal–Wallis test used.

Those using long‐term SL were less likely to have had someone else complete the answers on their behalf (by proxy), while outreach users were more likely to have used a proxy and less likely to have received no assistance. These differences need to be taken into account when describing the outcomes for older people using SL; users of different types of SL should not be treated as a homogenous group.

### Quality of life outcomes

Overall, 74% of the older people using SL rated their quality of life as ‘good’ or ‘very good’, 22% as ‘alright’, and 4% as ‘bad’ or ‘very bad’. The average SCRQoL score was 0.84, with a range of 0.22–1.00. Sixty‐eight per cent reported that they were ‘extremely’ or ‘very satisfied’ with their care and support, while a further 24% were ‘quite satisfied’.

Table 3 shows overall quality of life and SCRQoL for the sample, both overall and for each type of SL. Those using long‐term SL rated their quality of life more highly than those using day support or outreach, and also had better SCRQoL (numbers of those using respite were too small for differences to reach significance). The different aspects of SCRQoL were examined in terms of the percentage of people reporting the ideal situation or having no unmet need in that area of life. Those using long‐term/residential SL support were more likely to report having all needs met for control over daily life, social participation, occupation and dignity. In contrast, those using outreach support were less likely to report having all needs met in these areas. Those using day support were less likely to report having all needs met in the areas of occupation and food and drink; those using respite/short breaks were also less likely to report having needs met in the food and drink domain.

**Table 3:** Outcomes for older people using Shared Lives

	Long term	Day support	Respite/short breaks	Outreach	Overall	Test	*P*
Overall quality of life score (1–5)^a^							
Mean	1.42	2.06	2.29	2.37	1.95	*F* (3,141) = 12.29	<0.001
Standard deviation	0.64	0.89	0.76	0.93	0.91		
Range	1–3	1–4	1–3	1–5	1–5		
Missing	0	1	1	3	5		
SCRQoL score, weighted (−0.17 to 1.00)^b^							
Mean	0.95	0.78	0.80	0.77	0.84	*F* (3,131) = 12.32^c^	<0.001
Standard deviation	0.07	0.21	0.16	0.18	0.18		
Range	0.67–1.00	0.22–1.00	0.55–0.98	0.29–1.00	0.22–1.00		
Missing	4	3	2	6	15		
Aspects of SCRQoL (% ideal/no need)							
Control	98.1	76.5	87.5	65.5	80.5		<0.001^d^
Social participation	86.0	65.7	85.7	63.0	72.6		0.029^d^
Occupation	96.1	51.4	71.4	46.3	66.0		<0.001^d^
Food and drink	100.0	88.6	75.0	98.2	95.3		0.002^d^
Personal cleanliness and comfort	98.0	94.3	100.0	98.2	97.3		0.645^d^
Accommodation cleanliness and comfort	100.0	94.3	100.0	100.0	98.7		0.157^d^
Safety	98.0	88.6	85.7	85.5	90.5		0.081^d^
Dignity	100.0	87.9	100.00	79.6	90.1		0.005^d^

a Higher scores indicate poorer QoL.

b Higher scores indicate better SCRQoL.

c Bartlett's test of equal variances was statistically significant.

d Fisher's exact test was used, therefore no accompanying test statistic.

In order to discover whether the easy‐read element of the sample (many of whom were likely to have had a learning disability) was biasing these results, tests were re‐run without this group. Outcomes for people using long‐term SL were still statistically better for this smaller sample.

However, as described above, people using different types of SL support differ in terms of age, health and dependency and it is likely that this has an effect on their quality of life. In addition, people using long‐term/residential SL receive more of the support, which may also have an influence. To allow for this, multiple regression analyses were conducted to facilitate assessment of the unique effect of each variable on SCRQoL and on overall quality of life. The models analysed 123 and 116 cases respectively (the remainder were excluded through casewise deletion due to missing data on one or more variables). Table 4 shows the results for overall quality of life and SCRQoL. The models explained 37% of the variance in QoL scores and 45% of the variance in SCRQoL scores. Those who rated their health more poorly also reported a worse quality of life, as did those who experienced extreme anxiety or depression. Poorer SCRQoL was linked to reporting moderate pain or discomfort. Better SCRQoL was associated with being younger and with being female. Once these factors were controlled for, the type of SL support did not have a significant effect on QoL or SCRQoL scores.

**Table 4:** Factors associated with variations in outcome

	Overall quality of life^a^			SCRQoL^b^ coefficient (Beta)		
	Coefficient (Beta)	95% CI	*P*‐value	Coefficient (Beta)	95% CI	*P*‐value
Gender^c^	0.13 (0.07)	−0.40 to 0.14	0.334	0.07 (0.18)	0.00 to 0.13	0.037
Age	0.01 (0.05)	−0.01 to 0.03	0.617	−0.01 (−0.23)	−0.01 to 0.00	0.053
ADL score	−0.02 (−0.08)	−0.06 to 0.03	0.446	−0.01 (−0.11)	−0.01 to 0.00	0.298
Overall health	0.29 (0.31)	0.10 to 0.48	0.003	−0.03 (0.15)	−0.07 to 0.01	0.165
Pain/discomfort^d^						
Moderate	0.16 (0.09)	−0.12 to 0.44	0.249	−0.09 (−0.24)	−0.15 to −0.02	0.009
Extreme	−0.17 (−0.05)	−0.71 to 0.37	0.525	−0.02 (−0.02)	−0.12 to 0.08	0.726
Anxiety/depression^e^						
Moderate	0.21 (0.23)	−0.11 to 0.53	0.194	−0.02 (−0.06)	−0.10 to 0.06	0.609
Extreme	1.04 (0.23)	0.12 to 1.95	0.026	−0.16 (−0.18)	−0.35 to 0.02	0.084
Easy‐read version^f^	−0.08 (−0.04)	−0.42 to 0.26	0.631	−0.00 (−0.01)	−0.06 to 0.05	0.930
SL type^g^						
Day support	0.25 (0.12)	−0.18 to 0.68	0.258	−0.08 (−0.19)	−0.17 to 0.00	0.064
Respite/short breaks	0.25 (0.07)	−0.33 to 0.83	0.395	−0.02 (−0.02)	−0.14 to 0.11	0.773
Outreach	0.40 (0.21)	−0.73 to 2.14	0.118	−0.03 (−0.09)	−0.12 to 0.05	0.438
Overall model significance	*F* (12, 110) = 6.56, *P* < 0.001			*F* (12,103) = 9.93, *P* < 0.001		
*R* ^2^	0.37			0.45		

a Higher scores indicate poorer QoL.

b Higher scores indicate better SCRQoL.

c Male = 1, female = 0.

d Base = no pain/discomfort.

e Base = no anxiety/depression.

f Easy‐read = 1, main version = 0.

g Base = long term/residential.

### Comparison with older people using alternative care and support

A key aim of the survey was to facilitate comparison of outcomes of older SL users to older users of other social care services using data from the ASCS. The ASCS 2012 data set contains 164,569 cases. In order to reflect older people using SL, excluded were anyone: under 65; receiving nursing care; receiving equipment services from the LA but no other services; and those without information on outcomes. This resulted in a sample of over 30,000. Tests for differences between this subset of the ASCS 2012 and the SL data set indicated that the two samples differed on various background variables. There was a smaller proportion of females in the SL data set (χ^2^(1) = 22.45, *P* < 0.001). The SL sample were also younger, with a greater proportion of people aged between 65 and 74 and fewer over 85 (χ^2^(2) = 63.88, *P* < 0.001), and less dependent (*t*(28530) = −3.20, *P* = 0.0014). They experienced less pain or discomfort (χ^2^(2) = 8.92, *P* = 0.012) and rated their health more favourably (*z* = −3.317, *P* < 0.001).

To adjust for these differences, PSM was used to generate a matched sample and facilitate an unbiased comparison of outcomes. The conditional probability (propensity score) that any individual in the two samples (ASCS or SL) might be allocated to SL was estimated using logistic regression. The choice of conditioning variables in the logistic regression model is best informed by prior evidence and theory (Guo *et al*. 2006, Austin 2011); in this case, however, a lack of prior research evidence in the area and the small number of available background variables meant that choice was limited.

A number of models were tested to determine the most appropriate predictors of the probability of being allocated to SL. The aim was to achieve the best possible predictive value but minimise the exclusion of SL cases (a number of variables had missing values and thus cases were excluded). The predictive ability of all models tested was poor for SL cases, due in part to the small proportion that SL formed of the overall sample; however, this does not invalidate the match achieved. The final model included gender, age group, experience of pain, ability to get in and out of bed, ability to deal with paperwork, and overall health. PSM was conducted using STATA/PSMATCH2 based on nearest neighbour matching with calliper (1/4 SD) and without replacement (following Austin 2011). This produced a matched sample of 121 cases from the SL sample and 121 from the ASCS sample.

The matched SL sample was similar to the overall SL sample, differing only in terms of one indicator of dependency: ability to feed themselves (the non‐matched group were more likely to have difficulty with this task, *P* = 0.046, Fisher's exact). The matched ASCS sample included people using a mix of social care services including home care, day support and residential care, reflecting the varied nature of support available through SL. As they now ‘matched’ the SL users, this sample of the ASCS was on average younger (χ^2^(2) = 57.35, *P* < 0.001), less dependent (*t*(117.362) = −4.26, *P* < 0.001), in better health (*z* = −3.284, *P* = 0.001) and experienced less pain and discomfort than the overall sample (χ^2^(2) = 8.74, *P* = 0.01). There was a greater proportion of men (χ^2^(1) = 21.21, *P* < 0.001) and of people who completed the easy‐read questionnaire (χ^2^(1) = 18.13, *P* < 0.001) in the matched sample.

After matching, there was balance between the SL and ASCS samples on all the characteristics entered as variables in the model. However, there were significant differences between the groups in terms of the source of help received to complete the questionnaire. In addition, more people in the SL sample completed the easy‐read questionnaire (χ^2^(1) = 12.58, *P* < 0.001). It would not have been appropriate to use these variables in the logistic regression model, as they were not true background variables, but a feature of the questionnaire.

Table 5 shows the outcomes for the matched groups. There was a small but statistically significant difference in overall quality of life with SL users reporting better quality of life than those in the comparison group. However, there was no significant difference in overall SCRQoL scores for the two groups. For the individual domains of SCRQoL, the SL sample contained more people reporting the ‘ideal’ situation than simply having ‘no needs’ in the domains of food and drink and accommodation cleanliness. Results in the control, social participation and occupation domains were also in this direction, although did not reach statistical significance. Results in the safety domain, also non‐significant, showed a different pattern, with more people from the ASCS sample reporting the ‘ideal’ situation.

**Table 5:** Comparison of outcomes between SL and ASCS samples

	Shared Lives	ASCS sample	Test statistic	*P*
	*n* (%)	*n* (%)		
Overall quality of life score (1–5)				
Mean	1.94	2.18	*z* = 1.96	0.05
Standard deviation	0.91	0.97		
Range	1–5	1–5		
SCRQoL				
Mean	0.84	0.82	*t*(240) = −0.4856	0.63
Standard deviation	0.18	0.18		
Range	0.22–1.00	0.05–1.00		
Control				
No needs – ideal state	51 (42.2)	41 (33.9)	χ^2^(3) = 4.40	0.22
No needs	51 (42.2)	49 (40.5)		
Some needs	16 (13.2)	28 (23.1)		
High needs	3 (2.5)	3 (2.5)		
Social participation				
No needs – ideal state	51 (42.2)	48 (39.7)	χ^2^(3) = 1.32	0.72
No needs	42 (34.7)	49 (40.5)		
Some needs	24 (19.8)	19 (15.7)		
High needs	4 (3.3)	5 (4.1)		
Occupation				
No needs – ideal state	54 (44.6)	41 (33.9)	χ^2^(3) = 5.38	0.15
No needs	30 (24.8)	45 (37.2)		
Some needs	32 (26.5)	28 (23.1)		
High needs	5 (4.1)	7 (5.8)		
Dignity				
No needs – ideal state	72 (59.5)	67 (55.4)	χ^2^(3) = 4.20	0.24
No needs	36 (29.8)	46 (38)		
Some needs	13 (10.7)	7 (5.8)		
High needs	0	1 (0.8)		
Safety				
No needs – ideal state	83 (68.6)	89 (73.6)	χ^2^(3) = 2.47	0.48
No needs	27 (22.3)	27 (22.3)		
Some needs	9 (7.4)	4 (3.3)		
High needs	2 (1.7)	1 (0.8)		
Food and drink				
No needs – ideal state	96 (79.3)	79 (65.3)	χ^2^(3) = 8.74	0.03
No needs	20 (16.5)	38 (31.4)		
Some needs	5 (4.1)	3 (2.5)		
High needs	0	1 (0.8)		
Accommodation				
No needs – ideal state	96 (79.3)	80 (66.1)	χ^2^(3) = 5.94	0.05
No needs	23 (19)	28.9)		
Some needs	2 (1.7)	6 (5)		
High needs	0	0		
Personal cleanliness and comfort				
No needs – ideal state	74 (61.2)	74 (61.2)	χ^2^(3) = 1.11	0.78
No needs	44 (36.4)	41 (33.9)		
Some needs	2 (1.7)	4 (3.3)		
High needs	1 (0.8)	2 (1.7)		

## Discussion

The aim of this paper was to describe the outcomes of older people using SL support and to compare these to those of older people using alternative services, addressing the lack of evidence available about the potential of SL for older people. Although there are a number of caveats to our findings, the research offers some useful messages and provides a starting point for developing the evidence base for SL.

The study provides evidence that SL can deliver good outcomes, particularly for overall quality of life, echoing and building on the positive findings from more general research on SL (Fiedler 2005, NAAPS and IESE, 2009; Shared Lives Plus, 2014). The majority of SL users rated their overall quality of life highly and the average score for SCRQoL was also high, at a similar level to that found among non‐social care users (Netten *et al*., 2003), with those using long‐term placements reporting the best quality of life. For some respondents, the amount of support they received from SL would have been small – for example, a few hours a week for outreach support or a few weeks a year for short breaks. This means that the effect of SL on some aspects of their quality of life is likely to be minimal and outcomes may reflect other aspects of support than SL. Nonetheless, people using long‐term placements had consistently good outcomes across all domains of SCRQoL and it is these people where outcomes are most likely to reflect the support from SL. However, results of the regression models did suggest that it may be the differing characteristics of individuals using different models of SL, rather than the type of support itself, that was affecting quality of life outcomes.

There was some evidence that people using SL report a better quality of life than a similar group of older people using alternative care and support services. Although there was no difference in overall SCRQoL scores, ‘food and drink’ and ‘accommodation comfort and cleanliness’ were aspects of SCRQoL which appeared to be better for the SL users, with more people reporting the ‘ideal’ situation where needs are met to the optimum level. Results in some of the other domains including control, social participation and occupation also showed this pattern, although were not statistically significant. Responses to open questions included in the survey (described in Brookes *et al*. 2016) appeared to support these findings, with users of all types of SL support reporting that they valued the increased opportunities for social contact and getting involved in activities, many describing their SL carer as a friend or source of company and the value of feeling ‘part of a family’. Having choice over daily activities was seen as a key benefit of SL, as was the help to regain or maintain independence, both important elements of having control over daily life. It may be that the SL model of support could help address some of the negative aspects associated with older age such as loneliness and decreased social opportunities (Windle *et al*. 2011), and promote a sense of control. Indeed, it has been suggested that, for older people, control may be best achieved through relationships of trust with those that support them (Woolham 2015), something which SL services would seem ideally placed to facilitate.

The finding that users of SL rated their overall quality of life more highly than those using alternative care and support services, while there was no difference in SCRQoL, may indicate that SL offers some additional benefits not easily captured by the ASCOT measure, such as emotional support. Again, respondents’ comments about SL lend some support to this suggestion, linking the emotional support provided by the SL carer directly to improvement or maintenance of mental health and well‐being (Brookes *et al*. 2016).

### Limitations

There are a number of limitations to the analysis which need to be borne in mind when interpreting the results. The sample size was small, representing approximately 9% of older people using SL support, although it is the largest sample of older SL users achieved in research to date. Given that there is scarce information on the characteristics of SL users, it is difficult to determine how representative the sample is of the population, and care needs to be taken in generalising the findings. However, SL is generally considered to be suited to older people at the more able end of the spectrum who do not need nursing‐level care, and the characteristics of the sample in this study would seem to reflect that.

The sample was also complex: four types of SL support were represented, with users of each type having different characteristics. As there is so little evidence on SL, it was deemed important for the study to be as inclusive as possible but the small numbers in each subgroup meant more complex analysis was not possible; it would have been useful, for example to have considered the ‘outreach’ group separately given that this model differs from ‘traditional’ SL, and came only from one scheme. It was also not possible (given the small numbers) to analyse results by scheme; again a limitation to the findings presented here.

There are also limitations to the analysis used to compare the ASCS and SL samples. In the absence of a ‘real world’ comparison group, statistical matching techniques offer an alternative. However, the comparison made can only apply to the matched sample, not to those for whom there was no ‘match’ – in this case, the matched sample represents a tiny proportion of older people using social care and support services in 2011/2012. Although the older people drawn from the ASCS data set were using a range of types of care and support, they were younger, relatively able and in better health compared to the overall sample of older people. More information is needed about the characteristics of the older people using SL in order to be confident that this was comparing like with like.

## Conclusion

The research presented suggests that SL can provide a viable option alongside other forms of care and support for older people and one which reflects a number of current policy initiatives, offering choice and control and personalised support in a community setting (DH 2010, 2012, Care Act 2014). However, it is important to note that, although the sector is expanding, SL forms only a small part of social care and support provision for older people. Although there is a support for expansion of provision (Brookes & Callaghan, 2013), effort is needed to facilitate growth and raise awareness of the potential of SL. Further research to collect information on outcomes from a larger sample of older people would enable greater generalisability of findings, more sophisticated analysis of the different types of SL support and further comparative analysis.
